# Metabolic Deficiences Revealed in the Biotechnologically Important Model Bacterium *Escherichia coli* BL21(DE3)

**DOI:** 10.1371/journal.pone.0022830

**Published:** 2011-08-03

**Authors:** Constanze Pinske, Markus Bönn, Sara Krüger, Ute Lindenstrauß, R. Gary Sawers

**Affiliations:** 1 Institute for Microbiology, Martin-Luther University Halle-Wittenberg, Halle (Saale), Germany; 2 Institute of Computer Science, Martin-Luther University Halle-Wittenberg, Halle (Saale), Germany; Louisiana State University and A & M College, United States of America

## Abstract

The *Escherichia coli* B strain BL21(DE3) has had a profound impact on biotechnology through its use in the production of recombinant proteins. Little is understood, however, regarding the physiology of this important *E. coli* strain. We show here that BL21(DE3) totally lacks activity of the four [NiFe]-hydrogenases, the three molybdenum- and selenium-containing formate dehydrogenases and molybdenum-dependent nitrate reductase. Nevertheless, all of the structural genes necessary for the synthesis of the respective anaerobic metalloenzymes are present in the genome. However, the genes encoding the high-affinity molybdate transport system and the molybdenum-responsive transcriptional regulator ModE are absent from the genome. Moreover, BL21(DE3) has a nonsense mutation in the gene encoding the global oxygen-responsive transcriptional regulator FNR. The activities of the two hydrogen-oxidizing hydrogenases, therefore, could be restored to BL21(DE3) by supplementing the growth medium with high concentrations of Ni^2+^ (Ni^2+^-transport is FNR-dependent) or by introducing a wild-type copy of the *fnr* gene. Only combined addition of plasmid-encoded *fnr* and high concentrations of MoO_4_^2−^ ions could restore hydrogen production to BL21(DE3); however, to only 25–30% of a K-12 wildtype. We could show that limited hydrogen production from the enzyme complex responsible for formate-dependent hydrogen evolution was due solely to reduced activity of the formate dehydrogenase (FDH-H), not the hydrogenase component. The activity of the FNR-dependent formate dehydrogenase, FDH-N, could not be restored, even when the *fnr* gene and MoO_4_^2−^ were supplied; however, nitrate reductase activity could be recovered by combined addition of MoO_4_^2−^ and the *fnr* gene. This suggested that a further component specific for biosynthesis or activity of formate dehydrogenases H and N was missing. Re-introduction of the gene encoding ModE could only partially restore the activities of both enzymes. Taken together these results demonstrate that BL21(DE3) has major defects in anaerobic metabolism, metal ion transport and metalloprotein biosynthesis.

## Introduction

**Figure 1:**
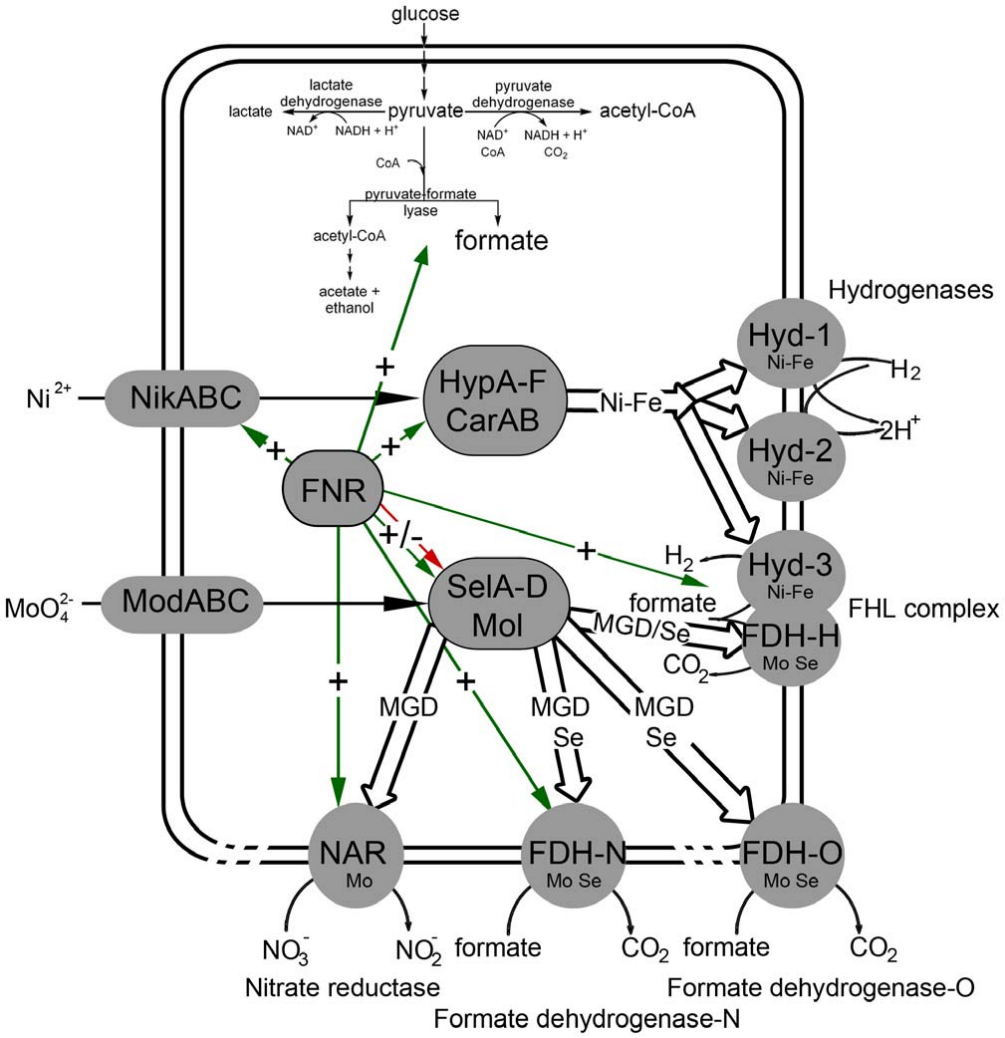
An overview of anaerobic hydrogen metabolism and nitrate respiration metabolism in *E. coli*. The metabolism of pyruvate under anaerobic conditions is shown in the upper portion of the Figure. The cellular locations of the three main [NiFe]-hydrogenases, the three molybdoselenium formate dehydrogenases and the principle nitrate reductase are shown, as are the transport systems for nickel and molybdate. The metal ion requirement and regulation with respect to the global regulator FNR are also indicated by arrows.

*Escherichia coli* is a facultative anaerobic enterobacterium that can grow both in the presence and in the absence of oxygen. When oxygen becomes limiting, *E. coli* can use nitrate or several alternative electron acceptors but if no exogenous electron acceptors are present it can resort to fermentation [1]. One of the key players in activating anaerobic gene expression is the global transcriptional regulator FNR (fumarate-nitrate regulator). FNR regulates, directly or indirectly, the expression of a very large number of genes and operons [2] whose products ensure that the optimal electron acceptor is utilized to allow maximum energy conservation. Many of the genes whose expression is induced by FNR encode complex metalloproteins, which have different metal cofactors in their active sites. Biosynthesis of these metal cofactors often requires the concerted action of a large number of accessory proteins. During nitrate respiration, for example, the FNR- and nitrate-dependent formate dehydrogenase (FDH) N and nitrate reductase (NAR) respiratory pathway is induced. Both enzymes have an array of iron-sulfur cluster, as well as the bis-molybdopterin guanidine dinucleotide (bis-MGD) cofactor [3]. Additionally, FDH-N requires co-translational insertion of selenocysteine in the polypeptide chain [4]. On the other hand, during fermentative growth *E. coli* has an active hydrogen metabolism and each of the three hydrogenases synthesized under these conditions has a [NiFe] cofactor. This cofactor must also be carefully assembled and inserted into the apo-enzyme [5]. Metals such as molybdenum and nickel must also be transported into the cell to allow synthesis of the appropriate metalloenzymes to occur. FNR exerts global control over many aspects of metalloprotein biosynthesis in *E. coli* and this is summarized in Fig. 1.

Hydrogenases catalyze the reversible oxidation of hydrogen and *E. coli* encodes four [NiFe]-hydrogenases (Hyd) in its genome, only three of which have been characterized [5]. All three enzymes are membrane-associated. Hyd-1 and Hyd-2 have their active site oriented toward the periplasm. Both enzymes contribute to generation of a proton gradient by oxidizing hydrogen on the periplasmic side of the cytoplasmic membrane and delivering the electrons from hydrogen oxidation directly into the respiratory chain. Hyd-3, together with the second molybdoselenoenzyme FDH-H, forms the hydrogen-evolving formate hydrogenlyase (FHL) complex, which has its active site localized towards the cytoplasm [6]. The substrate of the FHL complex is formate, which is generated during fermentation of sugar substrates such as glucose by the anaerobically inducible pyruvate formate-lyase (PflB) [1]. Formate is disproportionated into carbon dioxide and hydrogen by the FHL complex, thus off-setting acidification of the cytoplasm during fermentation.

The [NiFe]-cofactor in K-12 strains of *E. coli* such as MC4100 or MG1655 is common to all three hydrogenase enzymes. Thus, the majority of the accessory enzymes, referred to as Hyp proteins, required to synthesize this cofactor govern the biosynthesis of each hydrogenase [5], [7]. The Hyp proteins are involved in the synthesis of the non-proteinogenic ligands cyanide (CN^−^) and carbon monoxide (CO), which are coordinated to the iron atom in the [NiFe]-cofactor (for a review see [7]). The two CN^−^ ligands are derived from carbamoylphosphate (CP) [8], [9] while the source of the CO ligand is unclear. After synthesis and insertion of the modified iron atom into the large subunit precursor, nickel is then inserted through the action of HypA, HypB and SlyD [10]. The nickel required for biosynthesis of the active site is delivered by a specific ATP-binding cassette (ABC) transporter encoded by the FNR-regulated *nik* operon [11]. Lesions in the genes encoding the HypA and HypB enzymes, the nickel transporter, or indeed the global oxygen-responsive transcriptional regulator FNR can be phenotypically complemented by the addition of excess nickel ions to the medium [12].

Together with FDH-H, which is encoded by the *fdhF* gene [13], and FDH-N there is a third molybdenum- and selenium-containing formate dehydrogenase in *E. coli*, which is referred to as FDH-O (for a review see [14]). FDH-O is synthesized at low levels, preferentially in the presence of oxygen or nitrate [4], [15], [16]. Selenocysteine insertion in all three FDH enzymes occurs co-translationally with a sequence of reactions requiring the SelA, SelB and SelD proteins together with a specific tRNA^Sec^ encoded by *selC* [17]. Post-translational insertion of molybdenum in the form of the bis-molybdopterin guanidine dinucleotide (bis-MGD) cofactor [18] into the active site is also required for the activity of all three FDHs, and consequently hydrogen-evolving FHL complex activity.

While hydrogenase research in *E. coli* has focused on K-12 strains, comparatively little is known about hydrogen metabolism of B strains of *E. coli*. Research in the 1940's by Delbrück focused on *E. coli* B strains to study T phage function [19]. The commonly used BL21(DE3) strain was derived from an early isolate of *E. coli* B and was developed for T7 RNA polymerase-based gene expression after early isolation of a derivative carrying the DE3 prophage in its lambda attachment site [20], [21]. The DE3 prophage was introduced by P1 transduction from strain Bc258 and this was isolated as a non-reverting Gal^−^ derivative of Bc251, which had been obtained by UV treatment [22]. This UV treatment resulted in the loss of some genes important to metalloprotein biosynthesis, as described in this study.

Meanwhile BL21(DE3) has become established worldwide as a host for recombinant protein over-production. Despite this fact, it is surprising to note that the biology of BL21(DE3) is poorly characterized. Clearly, the strain does not have a wildtype phenotype because it has been shown that BL21(DE3) is unable to produce hydrogen gas [23], and indeed completely lacks hydrogenase activity, which we demonstrate in this study. This is despite BL21(DE3) having an apparently full complement of hydrogenase structural genes in its genome [24]. The genetic and metabolic reasons underlying this lack of hydrogen production are unclear. Here we demonstrate that the reasons for this metabolic deficiency of BL21(DE3) result from a lack of the global oxygen-responsive transcription factor FNR [11], [12], [25], [26], as well as severe deficiences in metalloprotein biosynthesis. This means that not only hydrogen metabolism but also nitrate respiration is compromised in the strain. These features have important implications for the use of BL21(DE3) and its derivatives in recombinant protein production, particularly for proteins of unknown function.

## Results

### BL21(DE3) is devoid of hydrogen metabolism

Only three of the four [NiFe]-hydrogenases (Hyd-1, Hyd-2 and Hyd-3) in *E. coli* K-12 strains are synthesized under standard laboratory conditions [6], [27]. Total hydrogenase enzyme activity in K-12 wildtype strains such as MC4100 or BW25113 can be readily determined by measuring hydrogen-dependent reduction of the artificial electron acceptor benzyl viologen (BV) 6,27. After anaerobic growth in buffered rich medium with glucose (TGYEP, pH 6.5) crude extracts derived from either strain had a total hydrogenase specific activity in the range of 3 U mg of protein^−1^ (Table 1). A crude extract derived from strain DHP-F2 (Δ*hypF*), which is unable to synthesize the HypF carbamoyltransferase essential for biosynthesis of the [NiFe]-cofactor of all three enzymes [28], lacked hydrogenase activity. Extracts of BL21(DE3) grown anaerobically in TGYEP were also devoid of hydrogen-dependent BV oxidoreductase activity (Table 1).

**Table 1:** Total Hydrogenase, hydrogen evolving formate hydrogen lyase activity and formate dehydrogenase-H (FDH-H).

Strain/Condition^1^	Specific Hydrogenase Activity in U mg protein^−1^ ± SD	Specific Hydrogen evolving Activity in mU mg protein^−1^ ± SD	Specific FDH-H Activity in U mg protein^−1^ ± SD
MC4100	3.01±0.59	28±20	0.42±0.08
CP971 (Δ*hycAI*)	0.14±0.08	<0.01	<0.01
CP971 (Δ*hycAI*)/p31hycA-I	8.12±0.40	19±1	0.05±0.01
DHP-F2 (Δ*hypF*)	<0.01	<0.01	0.04±0.01
BL21(DE3)	0.02±0.01 (0.004±0.003)	0.7±0.1 (<0.01)	<0.01 (<0.01)
BL21(DE3)/500 µM NiCl_2_	0.01±0.002	<0.01	<0.01
BL21(DE3)/15 mM formate	<0.01	<0.01	<0.01
BL21(DE3)/500 µM NiCl_2_/15 mM formate	0.01±0.01 (0.02±0.01)	<0.01 (3±2)	<0.01 (<0.01)
BL21(DE3)/pCH21 (*fnr* ^+^)	0.05±0.02 (2.22±0.44)	0.2±0.1 (8±3)	<0.01 (0.04±0.02)
BL21(DE3)/p1fnr	0.04±0.003 (2.84±0.69)	0.8±0.8 (7±2)	<0.01 (0.07±0.01)
BL21(DE3)/p10fnr	0.03±0.02 (0.02±0.01)	0.1±0.1 (0.3±0.3)	<0.01 (<0.01)
BL21(DE3)/p13fnr	0.03±0.02 (0.03±0.02)	<0.01 (0.6±1)	<0.01 (<0.01)
BL21(DE3)/p31hycA-I	<0.01	0.6±0.4	<0.01
BL21(DE3)/p31hycA-I/p1fnr	n. d.^2^	(2±0.5)	n. d.
BL21(DE3)/p7modE	0.02±0.01 (0.05±0.003)	<0.01 (0.5±0.6)	<0.01 (<0.01)
BL21(DE3)/p7modE/500 µM NiCl_2_/15 mM formate	0.01±0.003	<0.01	<0.01
BL21(DE3)/p7modE/p13fnr	0.03±0.003 (1.16±0.66)	<0.01 (6±2)	<0.01 (0.03±0.03)
BL21(DE3)/p7modE/p13fnr/500 µM NiCl_2_/15 mM formate	(3.71±1.92)	(15±3)	(0.08±0.01)
PB1000	0.13±0.21	5±3	<0.01
PB1000/500 µM NiCl_2_/15 mM formate	2.70±1.06	16±11	0.03±0.03
PB1000/pCH21	5.44±1.73	20±11	0.12±0.01
PB1000/p1fnr	6.16±1.30	34±6	0.50±0.06
PB1000/p10fnr	0.29±0.16	12±5	<0.01
PB1000/p13fnr	2.15±1.04 (2.59±1.20)	26±8 (21±2)	0.11±0.10 (0.10±0.04)

1 Cells were grown in TGYEP pH 6.5. Values in parenthesis were obtained when cells were grown in the presence of 1 mM sodium molybdate.

2 n. d. – not determined.

The activity of the FHL complex can be determined in whole cells by measuring hydrogen evolution [29]. While the FHL activity of MC4100 after fermentative growth with glucose attained levels of 28 mU mg of protein^−1^, BL21(DE3) failed to show any FHL activity, even after supplementation of the growth medium with formate, which is obligately required for the induction of the FHL complex [30]. DHP-F2 (Δ*hypF*) also lacked FHL activity, as anticipated and provided a negative control (Table 1).

### Bioinformatic analysis of the genes associated with hydrogen metabolism in the genome of BL21(DE3)

Initially, a total of 86 candidate genes that are known to have either a direct or indirect influence on hydrogenase activity in *E. coli* MC4100 or its sequenced counterpart MG1655 were chosen for comparison with the corresponding gene products in BL21(DE3). The deduced amino acid sequences of all 86 genes were examined to identify amino acid exchanges or deletions. Silent mutations that did not alter the amino acid sequence were ignored. Of the 86 candidate proteins examined with direct relevance to hydrogen metabolism only 42 proteins exhibited altogether 78 amino acid exchanges (missense mutations in the corresponding genes) and in 5 instances the corresponding genes were missing from the genome of BL21(DE3) completely (Table S1). Of this total carrying amino acid exchanges (or a nonsense mutation in the case of *fnr*), CarB, HypF, FNR, NikA, NikE, and NikD are the only putative candidates that could have a pleiotropic effect on hydrogenase activity resulting in a hydrogenase-negative phenotype.

### The activities of Hyd-1 and Hyd-2 in BL21(DE3) can be restored by nickel ion supplementation

**Figure 2:**
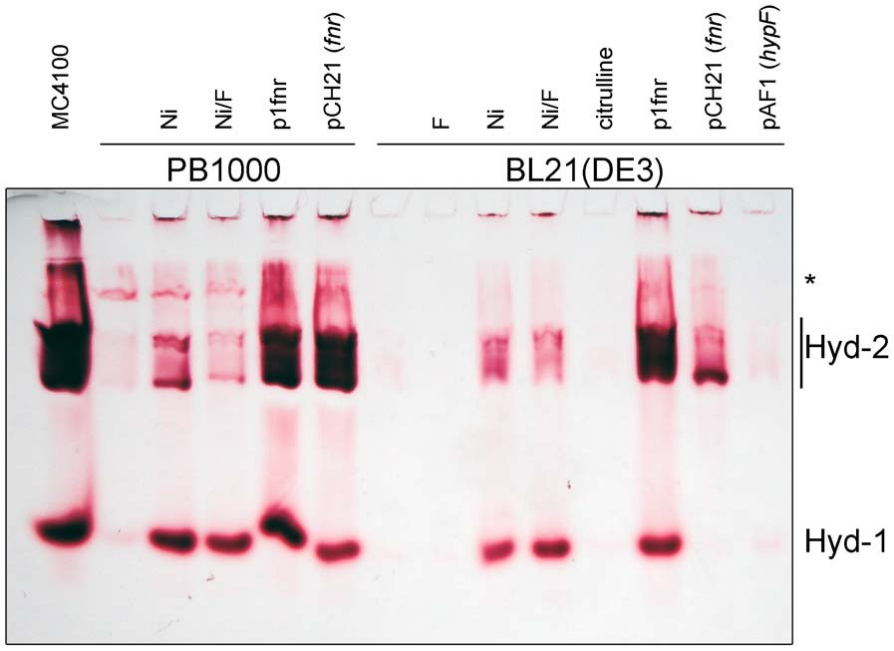
Hydrogenase 1 and 2 activity-staining after native-PAGE. Aliquots (25 µg of protein) of crude extracts derived from MC4100 (wild type), PB1000 (Δ*fnr*) and BL21(DE3) after anaerobic growth in TGYEP with or without supplementation of 500 µM nickel(II)-chloride (Ni), 15 mM formate (F) or 0.3 mM citrulline addition of plasmid-coded *fnr* (p1fnr, pCH21) and *hypF* (pAF1) were applied to 7.5% (w/v) native-PAGE. On the right hand the migration positions of Hyd-1 and Hyd-2 are given. The band designated with an asterisk is due to a side-reaction of FDH-O/FDH-N and this activity is hydrogenase-independent.

The *nik* operon codes for a specific ATP-binding cassette (ABC) transporter comprising a periplasmic binding protein NikA, the membrane components NikB and NikC, as well as the ATP-binding components NikD and NikE [31]. Defects in nickel-ion transport or HypA and HypB function, which are required for active hydrogenase biosynthesis can be phenotypically suppressed by addition of high concentrations of nickel ions to the growth medium [11], [32], [33] whereby non-specific Ni^2+^ ion uptake is mediated by the magnesium transport system [34]. The membrane components NikB and NikC showed no amino acid exchanges in BL21(DE3) compared to MG1655; however, the periplasmic binding protein NikA, as well as the ATP-binding components NikD and NikE had amino acid exchanges, with NikE having alterations in a total of six amino acids (see Table S1). In order to test first of all whether addition of high concentrations of Ni^2+^ ions to the growth medium could restore hydrogenase activity to BL21(DE3) we analysed hydrogen-dependent BV reduction (henceforth referred to as total hydrogenase activity) in crude extracts derived from BL21(DE3) grown anaerobically in the presence of 0.5 mM NiCl_2_. Only very low total hydrogenase activity could be determined and no hydrogen-evolving FHL activity could be measured (Table 1). Analysis of the activities of Hyd-1 and Hyd-2 after non-denaturing gel-electrophoretic separation of proteins in crude extracts of BL21(DE3) grown in the presence or absence of 0.5 mM NiCl_2_ (Fig. 2) revealed that while no activity could be visualised in BL21(DE3) grown without Ni^2+^ ion supplementation, addition of Ni^2+^ restored weak activities corresponding to Hyd-1 and Hyd-2; addition of formate, which was previously observed to result in increased Hyd-1 activity [6], had no effect on the activity band pattern (Fig. 2). This result suggested that nickel transport was indeed affected in BL21(DE3); however, addition of nickel at high concentrations could circumvent this phenotypic defect only partially.

To determine whether the mutations in *nikA*, *nikD* or *nikE* were responsible for the phenotypic defect in Ni^2+^, plasmids pJW3441, pJW3444 and pJW3445 (Keio collection; [35]), encoding NikA, NikD and NikE, respectively, were transformed into BL21(DE3) and total hydrogenase enzyme activity in crude extracts was determined (data not shown). None of the plasmids could restore hydrogenase activity, nor could activity bands corresponding to Hyd-1 or Hyd-2 be detected after native-PAGE (data not shown). It could be shown in complementation studies using the corresponding specific in-frame knockout mutants of *nikA*, *nikD* and *nikE* in the MG1655 derivative BW25113 (Keio collection; [35]) that their phenotype was hydrogenase-negative, that addition of 0.5 mM NiCl_2_ could restore hydrogenase activity to each mutant and that introduction of the respective plasmids encoding the *nikA*, *nikD* or *nikE* genes restored functional Hyd-1 and Hyd-2 either totally or partially (Fig. S1). This result demonstrates that the amino acid exchanges alone were not responsible for the defective nickel transport phenotype.

### Despite missense mutations in the respective genes carbamoylphosphate synthetase and HypF are functional in BL21(DE3)

CarB is the large subunit of the carbamoylphosphate synthetase providing carbamoylphosphate as the substrate for the cyanide ligand in the hydrogenase large subunits [36]. A defect in CarB function can be phenotypically suppressed by the addition of citrulline to the medium [36]. Although addition of citrulline did not restore hydrogenase activity to BL21(DE3) (Fig. 2), the fact that Ni^2+^ supplementation could partially restore hydrogenase function indicated that carbamoylphosphate synthetase must be functional in the bacterium and thus the two amino acid exchanges in CarB did not prevent enzyme function. Moreover, although addition of the *hypF* gene from MC4100 [37] did not restore active Hyd-1 or Hyd-2 (Fig. 2), by the same argument as brought above, the HypF of BL21(DE3) must nevertheless be functional.

HypF of BL21(DE3) shows 5 amino acid exchanges compared to MG1655 and 4 of these are also found in the HypF protein of *E. coli* O157:H7 (R51L/Y62H/K214N/S565P); however this protein retains its function [38]. As Hyd-1 and Hyd-2 activities can be restored in BL21(DE3) through supplementation of nickel ions without further addition of plasmid-encoded HypF it can be assumed that the 5^th^ amino acid exchange (D258E) has no influence on Hyd activities.

The ability to recover Hyd-2 enzyme activity by adding Ni^2+^ (see above) also obviated the missense mutations in the *hybD* and *hybF* genes (Table S1) as possible reasons why Hyd-2 was inactive in BL21(DE3). This was further confirmed by the fact that introduction of these genes from MG1655 failed to restore Hyd-2 activity to BL21(DE3) extracts (data not shown).

### BL21(DE3) is a *fnr* mutant

**Figure 3:**
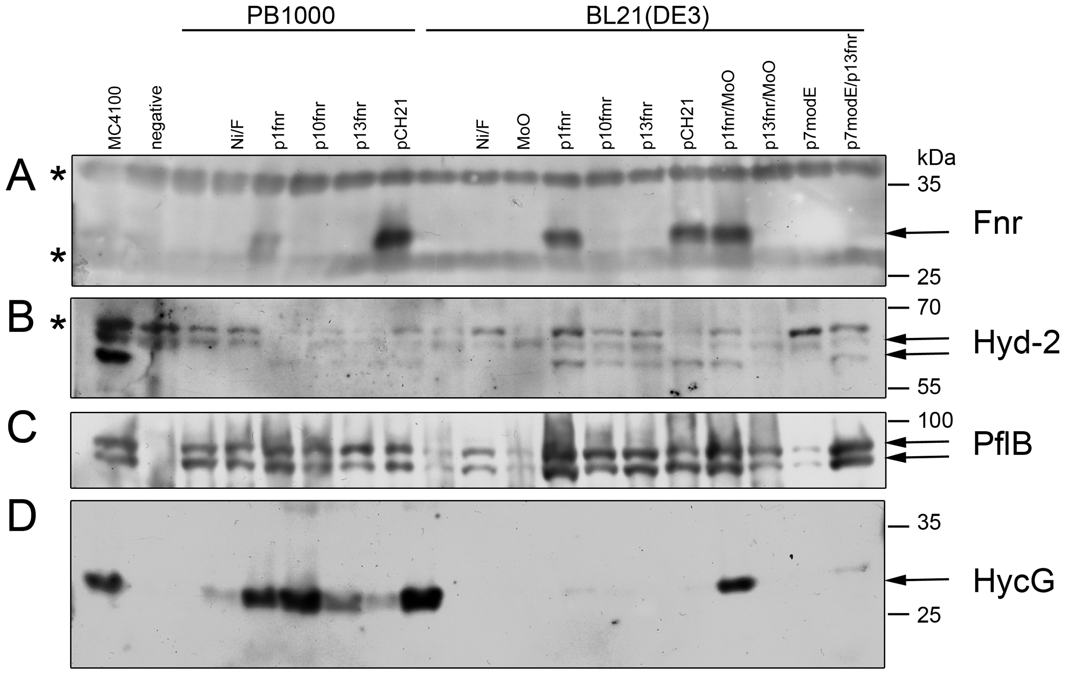
Western blot analysis of anaerobic enzymes in BL21(DE3). 25 µg Polypeptides in crude extracts derived from MC4100, PB1000 (Δ*fnr*), BL21(DE3) with and without supplementation of 500 µM nickel(II)-chloride (Ni), 15 mM formate (F), 1 mM sodium-molybdate (MoO) or addition of plasmid encoded *fnr* (p1fnr, p10fnr, p13fnr, pCH21) and *modE* (p7modE) after anaerobic growth in TGYEP, pH 6.5 were separated by 10% (w/v) SDS-PAGE and transferred to nitrocellulose membranes. The samples were treated with antiserum raised against **A:** FNR, **B:** Hyd-2 (the upper arrows represents precursor and the lower arrow mature form of the Hyd-2 large subunit), **C:** PflB (the arrows mark the two different migrating forms typical for active protein after contact with oxygen), **D:** HycG (the Hyd-3 small subunit). The lane indicating the negative control contains PB1000 (Δ*fnr*), DHP-F2 (Δ*hypF*), RM220 (Δ*pflAB*) and CP971 (Δ*hycAI*), from top to bottom, respectively. The asterisks signify unidentified cross-reacting species. On the right hand are given the sizes of the respective molecular mass marker (Prestained PageRuler, Fermentas).

Expression of the *nik* operon is dependent on the global transcriptional regulator FNR [12]. Furthermore, FNR also positively regulates the expression of the *hyp* operon [39], [40]. Analysis of the DNA sequence of the *fnr* gene in BL21(DE3) revealed a nonsense mutation (C→T transition) at codon 141, which resulted in an amber (UAG) stop codon [21]. Notably, many *E. coli* B strains carry this mutation [21], [24]. Western blot analysis of a crude extract derived from BL21(DE3) confirmed that full length FNR could not be detected (Fig. 3A).

We isolated a spontaneous *fnr* mutant (PB1000, Table 1) of MC4100 that carried a 3550 bp deletion from *insH-4* to the *fnr* gene and analysed the hydrogenase activity of this mutant. Total hydrogenase activity in extracts of PB1000 was reduced by >95% compared with the wildtype MC4100, FHL activity was reduced by 80% (Table 1) and the activity bands corresponding to Hyd-1 and Hyd-2 were barely detectable (Fig. 2). Introduction of the *fnr* gene on plasmid p1fnr into PB1000 restored total hydrogenase and FHL activities to wild type levels (Table 1). Transformation of BL21(DE3) with p1fnr resulted in a total hydrogenase specific activity of only 0.04 U mg^−1^ (compared with 3 U mg^−1^ for MC4100), while FHL activity was not restored at all by the plasmid. Although total hydrogenase activity was low, this activity nevertheless clearly represented fully active Hyd-1 and Hyd-2 under these growth conditions because activity-stained gels revealed active Hyd-1 and Hyd-2, which were restored to levels similar to those observed in K-12 wild type levels by p1fnr (Fig. 2). Because a *nik* operon mutation cannot be complemented by expression of the *fnr* gene [12] this allowed us to conclude that the missense mutations in the *nikA*, *nikD* and *nikE* genes of BL21(DE3) do not affect the function of the respective gene products.

The *fnr* gene on plasmid pCH21 is derived from MG1655 and includes the complete *fnr* regulatory region [41], while the *fnr* gene in p1fnr has a foreshortened and incomplete regulatory region with the consequence that there is 3 to 4-fold less FNR protein in MC4100 compared with MG1655 [42]. Transformation of BL21(DE3) with pCH21 also failed to restore hydrogen gas production or high level hydrogenase activity to the strain (Table 1). Surprisingly, however, although Hyd-2 enzyme activity could be visualised in crude extracts of BL21(DE3) transformed with pCH21 (Fig. 2), Hyd-1 was absent. Transformation of PB1000 with the same pCH21 restored total hydrogenase activity to wild type levels (Table 1 and Fig. 2). To ensure that plasmid-encoded FNR was synthesized in BL21(DE3), crude extracts of the transformed strain were analysed by Western blotting using anti-FNR antibodies. Plasmids p1fnr and pCH21 both resulted in high-level overproduction of the FNR protein (Fig. 3A). It was noted that the BL21(DE3) cells carrying pCH21 grew more slowly (μ = 0.56 h^−1^) than the plasmid-free strain, or BL21(DE3) transformed with pBR322 (μ = 0.77 h^−1^). In contrast, PB1000 (μ = 0.57 h^−1^) grew better when transformed with pCH21 (μ = 0.72 h^−1^). This might suggest that, because Hyd-1 synthesis is optimal in the stationary phase [43], [44], lack of induction of *hya* operon expression might account for this discrepancy.

Plasmids that had lower expression of the *fnr* gene compared with p1fnr or pCH21 were analysed to determine if too much FNR had a deleterious effect on expression of particular hydrogenase-related genes. The *fnr* gene from MC4100 was cloned without its regulatory region into a medium-copy vector (pBluescript SK+), delivering p10fnr, or into a low-copy vector (pACYC184), delivering p13fnr. The level of FNR protein synthesized in BL21(DE3) transformed with these two plasmids was similar to the wild type MC4100 or slightly less in the case of p13fnr (Fig. 3A). Total hydrogenase activity in the BL21(DE3) strain transformed with either of these plasmids was also very low and had a similar level of activity as observed with p1fnr or pCH21 (Table 1). Hyd-1 and Hyd-2 were barely detectable after activity-staining following native PAGE (data not shown). Thus, something else was limiting biosynthesis of active hydrogenases in BL21(DE3) and the effect was not caused by over-expression of *fnr*.

The presence of mature, processed large subunits of the hydrogenases can be used as an indicator as to whether the [NiFe]-cofactor maturation machinery is functional [7]. As expression of the *hyp* operon is FNR-dependent [39], we examined by Western blot analysis with antibodies raised against Hyd-2 whether transformation of BL21(DE3) with all four plasmids encoding FNR restored processing of the Hyd-2 large subunit precursor to the strain (Fig. 3B). The result confirmed that the *fnr* genes encoded on p10fnr and p13fnr were expressed and that the amount of FNR clearly did not limit Hyp protein synthesis or hydrogenase maturation activity because BL21(DE3) transformed with each plasmid showed clear processing of the Hyd-2 large subunit precursor.

### Lack of FNR and nickel transport does not explain why BL21(DE3) is devoid of FHL complex activity

The total hydrogenase activity in MC4100 grown under glucose fermentation conditions is 3.0 U mg^−1^, with the bulk of this activity being due to Hyd-3 activity, as can be seen from the activity in extracts of the Δ*hycAI* mutant, CP971, which has an activity of 0.14 U mg^−1^ (Table 1). Hyd-2 activity contributes less than 5% to the total hydrogenase activity and Hyd-1 activity contributes below 1% to the total under these conditions. This suggests that in BL21(DE3) only partial Hyd-1 and Hyd-2 activities were restored in the presence of *fnr* plasmids and Hyd-3 was inactive.

Exogenously added formate and nickel can phenotypically suppress the effect of *fnr* mutations on Hyd-3 and consequently FHL activity [6]. This is because FNR regulates PflB synthesis and consequently in a *fnr* mutant intracellular formate levels are reduced [45]. Western blot analysis of PflB levels in extracts of BL21(DE3) revealed that the protein was significantly reduced (Fig. 3C). Exogenous formate increased the level of PflB in the cell extracts and this is presumably due to build up of pyruvate, which induces *focApflB* operon expression [46]. All four plasmids encoding FNR also restored high-level PflB synthesis to BL21(DE3) (Fig. 3C).

Hydrogen gas production and total hydrogenase activity could be restored to near wild-type levels in PB1000 (Δ*fnr*) by supplementation of formate and Ni^2+^ to the growth medium (Table 1). In contrast, however, formate and Ni^2+^ supplementation alone could not restore Hyd-3 or FHL activity to BL21(DE3) (Table 1). Taken together, these findings indicate that, although BL21(DE3) has reduced levels of intracellular formate due to reduced PflB synthesis, this is not the only reason why an active FHL complex could not be synthesized.

Five of the *hyc* genes carry missense mutations (Table S1). To rule out that these limit Hyd-3 activity we cloned the complete *hyc* operon from the genome of MC4100 and introduced this on plasmid p31hycA-I into BL21(DE3) and determined total hydrogenase and FHL activities (Table 1). Although p31hycA-I complemented the Δ*hycA-I* mutation in CP971, when transformed into BL21(DE3) the plasmid failed to restore either Hyd-3 activity or hydrogen gas production (Table 1). Indeed, simultaneously introducing the *fnr* gene on p1fnr also failed to restore either activity. This result indicates that something else limits development of both FHL and Hyd-3 activity in BL21(DE3).

### Molybdenum uptake and metabolism are compromised in BL21(DE3)

As well as the requirement of [NiFe]-cofactor biosynthesis for hydrogen evolution there is also a necessity for co-translational selenocysteine incorporation and molybdenum cofactor biosynthesis for the FDH-H component of the FHL complex [47]. Further, the FdhD and FdhE proteins have been proposed to have chaperone-like functions and they are required for generation of functional FDH in *E. coli* [48].

Examination of the genome of BL21(DE3) revealed that five genes (*modABC*, *modE* and *modF*) are absent. The DE3 prophage insertion site is located directly in the region of the *mod* genes and it has been proposed that the deletion is due to an UV treatment in another strain and subsequent recombinant transfer by P1 transduction [21]. The ModABC proteins form the basis of the ABC transport system for the molybdate anion, while ModE is a molybdenum-responsive transcriptional regulator that represses expression of the *modABC* operon and activates expression of genes and operons whose products are either components of molybdoenzymes or are functional together with molybdoenzymes [49].

Initial experiments were conducted in which excess molybdate was added to cultures, whereby the molybdate anion can be taken up non-specifically by the sulphate transport system [50]. Molybdate had no effect on total hydrogenase activity when added alone or in combination with nickel and formate (Table 1). Total hydrogenase activity was, however, restored to levels approximating those of wild type K-12 strains when molybdate was added to BL21(DE3) transformed with pCH21 or p1fnr. These same cells also produced hydrogen at a level approximately 25% of the K-12 wildtype (Table 1).

Western Blot analyses revealed that although low amounts of HycG, the small subunit of Hyd-3, were detected in extracts of BL21(DE3) without addition of metal ions, HycG levels were significantly increased in BL21(DE3) transformed with p1fnr, but only when 1 mM molybdate was included in the growth medium (Fig. 3D).

Activity of the molybdenum cofactor-dependent FDH-H enzyme was partially recovered after growth of BL21(DE3) transformed with pCH21 or p1fnr, but only when 1 mM molybdate was included in the growth medium, which is consistent with the requirement of molybdate for active enzyme synthesis (Table 1). Nevertheless, this activity attained levels of at best only 10% of the activity determined in K-12 strains. This suggests that the amount of active FDH-H limits the activity of the H_2_-evolving FHL complex. Introduction of the *fdhF* gene on a plasmid had no effect on the FDH-H enzyme activity (data not shown), suggesting that maturation of the enzyme is what hinders a higher activity being attained.

ModE is a Mo-dependent transcriptional activator of genes and operons encoding many molybdenum cofactor-dependent enzymes [51]–[53]. Introduction of the *modE* gene on a multicopy plasmid into BL21(DE3) already containing the *fnr* gene on the low-copy number plasmid p13fnr, together with the addition of molybdate to the growth medium restored total hydrogenase activity to 30% of the K-12 wildtype and FHL activity to 20% of the K-12 wildtype level. This result demonstrates clearly that ModE regulates Hyd-3 biosynthesis [52] because omission of the p7modE plasmid resulted in recovery of neither high hydrogenase activity nor H_2_ production (Table 1). Finally, supplementation of the growth medium of BL21(DE3) transformed with p7modE and p13fnr with molybdate, nickel and formate resulted in H_2_ production that was roughly 50% that of the K-12 strains (Table 1). Moreover, FDH-H polypeptide could be detected in extracts of this strain, indicating that selenocysteine incorporation [54] was functional in BL21(DE3); further addition of selenite or selenate to the growth medium failed to increase formate dehydrogenase enzyme activity further, suggesting that transport of the anion was not limiting (data not shown).

### BL21(DE3) derivatives from other sources also have a hydrogenase-negative phenotype

To ensure that the phenotypes identified here to be associated with BL21(DE3) are not restricted to a strain from a particular source, we analyzed the ability of two BL21(DE3) derivatives from other sources for their ability to generate hydrogen. The Rosetta strain of BL21(DE3) (Novagen) has optimized codon usage for heterologous protein overproduction, while C41(DE3) was isolated specifically for the recombinant overproduction of membrane proteins [55] and is a derivative of the BL21(DE3) strain originally used by Studier and Moffatt [14]. Both BL21(DE3) were transformed with plasmid pCH21 carrying the *fnr* gene and were grown in the presence and absence of 1 mM molybdate. Hydrogen was only produced by the strains when the additional copies of the *fnr* gene were introduced and molybdate was present in the growth medium (Table S2), indicating that other BL21(DE3) derivatives share the metabolic defects identified for BL21(DE3) obtained from Novagen.

### BL21(DE3) cannot respire with nitrate

Three further bis-MGD-containing enzymes present in *E. coli* and which influence anaerobic growth are nitrate reductase (NAR), and FDH-N and FDH-O, the latter two are also selenoenzymes [14]. FDH-N and NAR are inducible in the presence of nitrate and allow the bacterium to respire anaerobically with nitrate as electron acceptor [56]. FDH-O is phylogenetically related to FDH-N; however, the enzyme is synthesized at a low level both aerobically as well an anaerobically in the presence of nitrate [57], [58].

**Figure 4:**
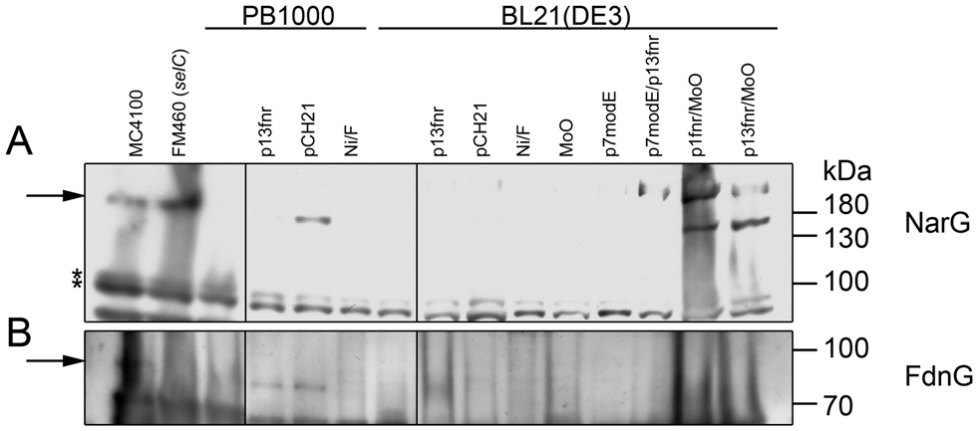
Western blot analysis of the large subunits of NAR and FDH-N. Crude extracts (25 µg protein) of MC4100, FM460 (Δ*selC*), PB1000 and BL21(DE3) bearing plasmids p13fnr (*fnr* ^+^), pCH21 (*fnr* ^+^), p7modE (*modE* ^+^) or supplemented with 500 µM nickel(II)-chloride (Ni), 15 mM formate (F) or 1 mM molybdate (MoO), when indicated were separated on 10% (w/v) SDS-PAGE after anaerobic growth in TGYEP, pH 6.5 with 100 mM potassium-nitrate. and treated with antiserum raised against **A:** Nar or **B:** FdnG.

Crude extracts of BL21(DE3) grown anaerobically in rich medium in the presence of nitrate exhibited neither FDH-N nor NAR enzyme activity (Table 2). In contrast, extracts derived from the K-12 strain MC4100 grown under the same conditions had high activities of both enzymes. After transformation of BL21(DE3) with pCH21 or p1fnr neither enzyme activity could be detected. Addition of sodium molybdate to these cultures restored NAR activity but, surprisingly, not the activity of FDH-N (Table 2). Western blots revealed that the large subunit of the NAR enzyme (NarG) was only detected in the presence of multicopy *fnr* and molybdate (Fig. 4A).

**Table 2:** Specific activities of Nitrate reductase and Formate dehydrogenase N (FDH-N).

Strain/Condition^1^	Specific Nitrate reductase activity in U mg protein^−1^ ± standard deviation	Specific FDH-N Activity in U mg protein^−1^ ± standard deviation
MC4100	0.60±0.28	0.39±0.12
BL21(DE3)	0.01±0.02 (<0.01)	<0.01 (<0.01)
BL21(DE3)/pCH21 (*fnr* ^+^)	<0.01 (0.54±0.17)	<0.01 (<0.01)
BL21(DE3)/p1fnr	0.02±0.02 (0.40±0.23)	<0.01 (<0.01)
BL21(DE3)/p10fnr	<0.01 (0.08±0.08)	<0.01 (0.01±0.01)
BL21(DE3)/p13fnr	<0.01 (0.11±0.08)	<0.01 (<0.01)
BL21(DE3)/p7modE	<0.01 (<0.01)	<0.01 (0.05±0.04)
BL21(DE3)/p7modE/p13fnr	<0.01 (<0.01)	<0.01 (0.04±0.03)
BL21(DE3)/pJW3563 (*selB* ^+^)/p1fnr	0.02±0.01 (0.11±0.24)	<0.01 (0.01±0.002)
BL21(DE3)/pJW1753 (*selD* ^+^)/p1fnr	0.03±0.01 (0.13±0.12)	<0.01 (<0.01)
PB1000	0.01±0.01	0.04±0.02
PB1000/pCH21	0.59±0.15	0.19±0.03
PB1000/p1fnr	0.47±0.15	0.24±0.05
PB1000/p10fnr	0.02±0.02	0.16±0.09
PB1000/p13fnr	0.16±0.06 (0.13±0.01)	0.15±0.08 (0.16±0.06)

1 Cells were grown in TGYEP pH 6.5 supplemented with 100 mM KNO_3_. Values in parenthesis were obtained when cells were grown in the presence of 1 mM sodium molybdate.

A low activity of the nitrate-inducible FDH-N, attaining levels of 10% of K-12 strains, was only measurable in the presence of plasmids encoding FNR and ModE and when molybdate was added to the growth medium (Table 2). FDH-N activity could not be restored to this strain by introducing functional *selB*, *selD*, *fdhD* or *fdhE* genes on plasmids (Table 2 and data not shown). No condition could be identified that resulted in high FDH-N enzyme activity, which clearly would limit growth of BL21(DE3) by nitrate respiration using formate as electron donor. Although a FDH-N activity of 10% of the K-12 wild MC4100 could be recorded when the *fnr* and *modE* genes were introduced into BL21(DE3) and molybdate was added to the growth medium (Table 2), the large subunit of FDH-N was below the threshold of detection by Western blotting (Fig. 4B).

**Figure 5:**
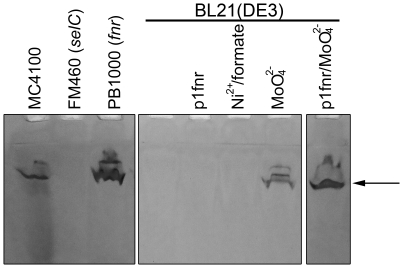
The activity of FDH-O in BL21(DE3) is restored with high concentrations of molybdate. Crude extracts (25 µg of protein) of the various strains indicated were separated in non-denaturing PAGE and stained specifically for FDH-O activity as described in the methods section. The arrow indicates the position of the active FDH-O enzyme.

The activity of FDH-O can be visualized after native-PAGE using formate as a substrate and nitroblue tetrazolium (NBT) as an artificial electron acceptor [59]. No activity of this enzyme could be detected in extracts of BL21(DE3) grown anaerobically (Fig. 5). Bioinformatic analysis of the genes encoding *fdoGHI* revealed that no missense mutations were present (Table S1), indicating that this could not be the reason for the lack of enzyme activity. However, supplementation of the growth medium with molybdate restored FDH-O enzyme activity. Addition of the *fnr* gene into BL21(DE3) on a plasmid had no effect, which is in accord with the *fdoGHI* operon not being FNR-dependent [15]. The restoration of FDH-O activity also confirmed that the *sel* and *fdhD* and *fdhE* gene products of BL21(DE3) have sufficient activity to allow synthesis of active FDH-O.

## Discussion

Although BL21(DE3) is an *fnr* mutant this is not the sole explanation for complete lack of hydrogen metabolism in the strain. For example, while the spontaneously isolated *fnr* deletion mutant, PB1000, of the K-12 strain MC4100 described in this study has significantly reduced hydrogenase activity, nevertheless, a low activity was still measurable. In contrast, no hydrogenase activity whatsoever in extracts or whole cells of BL21(DE3) could be detected, despite a full complement of structural genes being present in the genome [24]. Analysis of the deduced structural gene products revealed that, while Hyd-1 lacks amino acid exchanges, components of Hyd-2 carry some substitutions. In particular, however, a considerably higher number of mutations in Hyd-3 components could be identified. Nevertheless, all of these amino acid substitutions could be ruled out as the reasons for the lack of hydrogenase activity. Rather, in the cases of Hyd-1 and Hyd-2, the lack of FNR caused restricted nickel import with the consequence that the biosynthesis of the active site of these enzymes could not be completed. In the case of Hyd-3 neither nickel nor FNR augmentation was enough to restore enzyme activity. This proved to be, at least in part, due to impaired molybdenum transport activity as well as due to the lack of the *modE* gene. ModE is a molybdenum-responsive transcriptional regulator that was identified to be required, along with FHLA and formate, to allow maximal expression of the *fdhF* gene and the *hyc* operon [52], [60]. The observed partial dependence on molybdate and ModE for FHL biosynthesis could be verified in this study.

Nevertheless, although Hyd-3 activity could be restored to levels equivalent to K-12 wildtype strains grown under the same conditions, it was not possible to restore the hydrogen evolution activity of the FHL complex to wildtype levels. This proved to be due to a limitation in the activity of FDH-H, which could only be recovered to maximally 10–15% of that measured for MC4100. Analysis of FDH-N, which, along with nitrate reductase, is induced in the presence of nitrate and allows *E. coli* to grow by nitrate respiration, was also completely inactive in extracts of BL21(DE3) unless a combination of FNR, ModE and molybdate was supplied to the strain. Nevertheless, like FDH-H, which was also induced under these conditions, the activity reached maximally 15% of K-12 wild type. That nitrate reductase activity was induced to K-12 wildtype levels by the introduction of *fnr* on a plasmid, along with supplementation of the growth medium with molybdate, indicated that the limitation in the activities of both FDH selenomolybdoenzymes was not in bis-MGD biosynthesis or insertion or in gene expression of the corresponding structural genes. Moreover, the fact that the third FDH-O could be actively synthesized simply by adding only molybdate confirmed that selenocysteine biosynthesis and insertion is not compromised in BL21(DE3); it should be noted, however, that the amounts of the FDH-O enzyme in MC4100 extracts are considerably lower than those of the other two FDHs [58], [61]. Biosynthesis of both FDH-H and FDH-N requires the private chaperones FdhD and FdhE [48], [62], [63]. Although the corresponding genes are present in the genome of BL21(DE3) both have single amino acid deletions, which could influence the efficiency with which both enzymes function. Additionally, both enzymes are iron-sulfur proteins and interact with components of the iron-sulfur biosynthetic machinery [64]. It is therefore theoretically possible that insufficient supply of iron could compromise the activities of these proteins. Notably, however, the gene encoding the IscR regulator and the iron-sulfur cluster insertion protein IscA do not carry any mutations when compared with their MG1655 counterparts (Table S1). Nevertheless, a further in-depth study will be required to determine whether the iron-sulfur biogenesis machinery is fully functional in BL21(DE3).

As well as having defects in nickel enzyme biosynthesis through the lack of FNR, molybdenum acquisition is also compromised, as is cobalt uptake through the nonsense mutation in the *btuB* gene [21]. Clearly, the extent to which maturation of other metalloproteins with further metal requirements is compromised in BL21(DE3) was outside the scope of this study. Nevertheless, this is an important issue to address in future metalloproteomic analyses using BL21(DE3). Moreover, it should be emphasized that all derivatives of the BL21(DE3) strain analyzed in this study lack a functional *fnr* gene, are deleted in the genes encoding molybdenum transport function and as we could demonstrate here have a similar hydrogenase-negative phenotype to the Novogen strain of BL21(DE3).

The results of metalloproteomic studies have estimated that at least 40% of all proteins in all organisms are metalloproteins [65]. This is likely to be a conservative estimate because through the development of new high-throughput tandem mass and inductively coupled plasma mass spectrometry techniques combined with classical protein purification new, previously undiscovered metalloenzymes, (including new nickel- and molybdenum-containing enzymes) with as yet unknown functions, are being discovered [66]. The inevitable transfer of the genes encoding these novel metalloproteins into recombinant expression hosts, such as BL21(DE3), for large-scale protein production necessitates an appreciation of the limits of an expression system, particularly when trying to identify new protein functions with previously uncharacterized metal ion cofactors.

The influence of the *fnr* mutation on growth and metabolism of BL21(DE3) also should not be underestimated. Large-scale transcriptome studies have shown that FNR controls, directly or indirectly, the expression of at least one third of all the genes in the *E. coli* K-12 genome and this includes a large contingent of ‘aerobic’ genes [67], [68]. Moreover, although BL21(DE3) is usually cultured for recombinant protein production in rich medium in the presence of air, it is very difficult, even in shake flasks, to supply *E. coli* with sufficient oxygen when growing in rich medium to maintain aerobiosis and cultures inevitably become oxygen-limited very quickly [69].

*E. coli* B was isolated, probably as a commensal of the human intestinal tract, in the early part of the 20^th^ century [22]. Hydrogen generation by commensal or pathogenic strains could pose an evolutionary disadvantage in the host. For example, neither *Yersinia pestis*, *Shigella flexneri* nor *S. dysenteriae* produce hydrogen gas [70]; all three are pathogens. Moreover, the human pathogenic strain *Salmonella enterica* does not release hydrogen gas, because the uptake hydrogenase is extremely efficient [71]. On the other hand, hydrogen oxidation possibly provides a growth advantage for pathogenic bacteria. It has been shown for *Helicobacter pylori* that in the presence of hydrogen, growth and colonization of the stomach was improved [72] while *Campylobacter* spp. are also able to oxidize hydrogen [73]. *E. coli* strain BL21(DE3) is not listed as a pathogenic strain; however, being closely related to pathogenic strains like O157:H7 it is not surprising that evolutionary hydrogen gas production was perhaps counter-selected as can be deduced from the accumulation of amino acid exchanges within the FHL complex. The loss of FNR apparently occurred before *E. coli* B strains entered the laboratory [22]. We noted that when the *fnr* gene was reintroduced into BL21(DE3) anaerobic growth slowed, which contrasts with what is normally observed with K-12 strains [74]. The reasons for the better growth of BL21(DE3) lacking FNR are intriguing and worthy of further elucidation. The recent demonstration [75] that synthesis of the other global redox-sensing regulator ArcA [76] [22] is possibly limiting in BL21(DE3) might also impact significantly on these metabolic deficiences.

## Methods

### Strains and growth conditions

All strains and plasmids used in this study are listed in Table 3. Aerobic growth was carried out in LB medium [77] in shaking cultures at 37°C. For growth on agar plates media were solidified by inclusion of 1.5% (w/v) agar. For qualitative hydrogen gas production 10 ml of LB medium with 0.8% (w/v) glucose with Durham tubes were used as described [78]. Anaerobic growths to determine hydrogenase activity were done in 100 ml of TGYEP, pH 6.5 as described [79] and for growth curves the optical density at 600 nm was measured in a NOVOStar plate-reader (BMG Labtech, Germany) in sealed 96-well microtiter plates at 37°C. Anaerobic growth in microtiter plates was verified by native-PAGE with subsequent staining for hydrogenase activity [27]. When needed, kanamycin, chloramphenicol or ampicillin was added to the medium to final concentrations of 50, 12.5 and 100 µg ml^−1^, respectively. Where indicated, addition of nickel(II)-chloride was done to a final concentration of 500 µM, of sodium molybdate (MoO_4_^2−^) to 1 mM, of formate to 15 mM and of KNO_3_ to 100 mM.

**Table 3:** Strains and plasmids used in this study.

Strains	Genotype	Reference
MC4100	*F*- *araD139* Δ(*argF-lac*)*U169 ptsF25 deoC1 relA1 flbB5301 rspL150*-	[89]
DHP-F2	MC4100 Δ*hypF*	[28]
JW1753	BW25113 Δ*selD*	National BioResource Project (NBRP) – *E. coli* at National Institute of Genetics (NIG)
JW3563	BW25113 Δ*selB*	NBRP-*E.coli* at NIG
BL21(DE3)	*F* ^−^ *ompT gal dcm lon hsdS_B_*(*r_B_* ^−^ *m_B_* ^−^) λ(DE3 [*lacI lacUV5-T7 gene 1 ind1 sam7 nin5*])	Novagen, USA
Rosetta(DE3) pLysS	*F* ^−^ *ompT hsdS_B_* (*r_B_^−^ m_B_^−^*) *gal dcm* λ(*DE3* [*lacI lacUV5-T7 gene 1 ind1 sam7 nin5*]) pLysSRARE	Novagen, USA
C41(DE3)	*F* ^−^ *ompT gal dcm hsdS_B_* (*r_B_^−^ m_B_^−^*) λ(DE3), Like BL21(DE3) but with an uncharacterized mutation affecting membrane protein synthesis	[55]
CP938	BW25113 Δ*hycA-I*::Kan	This work
CP971	MC4100 Δ*hycA-I*::Kan	This work
PB1000	MC4100 Δ*purT* Δ*purU* Δ*insH4-fnr*	This work
FM460	MC4100 Δ(*selC*)*400*::Kan	[58]
RM220	MC4100 Δ*pflB*-*pflA*	[90]
Plasmids		
pAF1	Cm^R^, *hypF*	[37]
pJW3563	ASKA Clone(-) *selB*	[35]
pJW1753	ASKA Clone(-) *selD*	[35]
pJW3441	ASKA Clone(-) *nikA*	[35]
pJW2444	ASKA Clone(-) *nikD*	[35]
pJW3445	ASKA Clone(-) *nikE*	[35]
pCH21	Ap^R^, Cm^R^, *fnr*	[41]
pBR322	cloning vector	[91]
p1fnr	genomic *E. coli* SauIIIA fragments in pBR322, containing *fnr*	[37], This work
p10fnr	pBluescript SK(+) containing *fnr* in HindIII and BamHI site; Amp^R^	This work
p13fnr	pACYC184 containing *fnr* in HindIII and BamHI site; Cm^R^	This work
pACYCM	pACYC184 with A1845T exchange in *tetA*; Cm^R^	This work
p31hycAI	8365 bp MluI insert from *hycAI* into pACYCM; withershins *tetA*; Cm^R^	This work
p7modE	pBluescript SK(+) containing *modE* in BamHI and EcoRI site; Amp^R^	This work
p2modE	pACYC184 containing *modE* from p7modE in BamHI and EcoRV site; Cm^R^	This work

### Genetic manipulations and plasmid construction

Transformation of plasmids and recombinant DNA work was done as described [80]. Construction of the reference strain CP938 (BW25113 Δ*hycA-I*) was done as described in [81] with the strain BW25113 carrying plasmid pKD46. PCR with Phusion DNA polymerase (Finnzymes, Germany) was conducted using the chloramphenicol resistance cassette from plasmid pKD4 as template and the oligonucleotides hycA_5′ 5′-GCT TAA AGC TGG CAT CTC TGT TAA ACG GGT AAC CTG ACA *CCA TGG TCC ATA TGA ATA TCC TCC*-3′ and hycI_3′ 5′-CCC ATC AAG AAC ATC CCT GTC CTG ATT CCT TAA TGA AAA A*GC GAT TGT GTA GGC TGG AGC T*-3′ (Metabion, Germany). The replacement of the *hyc* operon with the chloramphenicol-resistance cassette was verified by PCR amplification with oligonucleotides outside of the operon (hyp_K 5′-CTC *GGA TCC* TGT CAC CAT GAC ACT GTG GA-3′ and hycI_K 5′-CAG CGC ATC GGG CAA TTT AG-3′). The *hyc*-operon deletion allele was then transduced by phage P1*kc* transduction [77] into MC4100 resulting in strain CP971.

Three different plasmids containing the *fnr* gene from MC4100 were isolated or constructed for complementation of the *fnr* mutations in PB1000 and BL21(DE3). The *fnr* gene present on pCH21 [41] was also used for complementation analyses. Plasmid p1fnr was isolated from a gene library derived from MC4100 genomic DNA [82] by complementing the *fnr* mutation in PB1000 and screening for restoration of hydrogen-dependent reduction of benzyl viologen activity [83]. The DNA insert in plasmid p1fnr encompassed the *insH-4*, *ynaJ*, *uspE* and *fnr* genes (4.3 kb insert). Amplification of the *fnr* gene from MC4100 genomic DNA was done with Phusion DNA polymerase and oligonucleotides Fnr_HindIII_FW 5′-GTG *AAG CTT* ATG ATC CCG GAA AAG CGA ATT A-3′ and Fnr_BamHI_RW 5′-GTG *GGA TCC* TCA GGC AAC GTT ACG CGT ATG-3′. The resulting 765 bp DNA fragment was digested with HindIII and BanHI and ligated into pre-digested pBluescript SK(+) and pACYC184 vectors resulting in plasmids p10fnr and p13fnr, respectively. The cloning of the *modE* gene from MC4100 was performed in a similar manner except that the oligonucleotides modE_FW_BamHI 5′-CGC *GGA TCC* ATG CAG GCC GAA ATC CTT C-3′ and modE_RW_EcoRI 5′-CGC *GAA TTC* TTA GCA CAG CGT GGC GAT AAT C-3′ were used. The resulting 807 bp DNA fragment was digested with BamHI and EcoRI and ligated into BamHI-HindIII-digested pBluescript SK(+) resulting in p7modeE. The *modE* gene was subcloned into pACYC184 via BamHI and EcoRV digestion resulting in the plasmid p2modE. The DNA sequences of the cloned genes were verified (Seqlab). The cloning of the *hycA*-*I* operon was done by direct digestion of genomic DNA from strain MC4100 with MluI, which resulted in an approximate 8500 bp DNA fragment that was excised from an agarose gel and ligated into a modified pACYC184 vector (pACYCM). To generate pACYCM, the *tetA* gene of pACYC184 was modified to include a MluI restriction site by exchanging A1845T with the oligonucleotides pACYC184_A1845T_FW 5′-CTA TCG ACT ACG CG**T** TCA TGG CGA CCA CAC-3′ and pACYC184_A1845T_RW 5′-GTG TGG TCG CCA TGA **A**CG CGT AGT CGA TAG-3′ using the QuickChange site-directed mutagenesis procedure (Stratagene). The orientation of the insert in p31hycA-I with respect to the *tetA* gene was verified by PCR and partial DNA sequence analysis. The functionality of the insert was tested by transforming p31hycA-I into strain CP971 (Δ*hycA-I*), which restored hydrogen gas production.

### Determination of enzyme activities

Dye overlay methods for colony screening were applied as has been described for formate dehydrogenase activity [83] with 0.5 mM benzyl viologen and a hydrogen atmosphere for hydrogenase activity or 2.5 mM benzyl viologen and 250 mM formate for formate dehydrogenase activity after anaerobic growth on agar plates in GasPak anaerobic jars (Oxoid, UK).

Anaerobic cultures were harvested at an OD_600 nm_ of approximately 0.8. Cells from cultures were harvested by centrifugation at 4000× g for 10 min at 4°C. The cell pellet was resuspended in 1% (v/v) of the culture volume of 50 mM MOPS buffer pH 7.0 and lysed on ice by sonication (30 W power for 5 min with 0.5 s pulses). Unbroken cells and cell debris were removed by centrifugation for 15 min at 10,000× g and 4°C and the supernatant was carefully decanted and used as the crude cell extract. Total enzyme activities were measured using 1 cm path-length anaerobic cuvettes in an Uvicon 900 dual-wavelength spectrophotometer according to [27] except that the buffer used was 50 mM MOPS buffer, pH 7.0 with 4 mM benzyl viologen. To determine hydrogenase activity the gas phase of the cuvettes was replaced with 100% hydrogen gas and the detection wavelength used was 578 nm and an E_M, 578_ value of 8,600 M^−1^ cm^−1^ was assumed for reduced benzyl viologen. Formate dehydrogenase H (FDH-H) activity was measured under the same conditions except that the cuvettes were flushed with nitrogen. The reaction was started by the addition 30 mM sodium formate. Nitrate reductase (NAR) enzyme activity was measured using 0.4 mM benzyl viologen reduced to an OD_600_ of 2 with freshly prepared 10 mM sodium dithionite solution. The reaction was started by the addition of 9 mM sodium nitrate as described [84] with an E_M,600 nm_ value of 7,400 M^−1^ cm^−1^ assumed for reduced benzyl viologen. Formate dehydrogenase N (FDH-N) activity was measured using final concentrations of 75 µM 2,6-dichlorophenolindophenol (DCPIP) and 288 µM phenazine methosulfate (PMS) and the reaction was started by the addition of 40 mM formate [84]. An E_M, 600 nm_ of 20,000 M^−1^ cm^−1^ for oxidised DCPIP was assumed. The specific activity of the FHL complex, measured as hydrogen evolution, was assayed in whole cells as described [29]. One unit of activity was defined as the oxidation of 1 µmol of the respective substrate per min. All activities were determined from 3 independent cultures. Protein concentration was determined [85] with bovine serum albumin as standard.

### Polyacrylamide gel electrophoresis and in-gel hydrogenase activity-staining

For Western blot analysis, aliquots of 50 µg protein from crude extracts were separated in 10 or 12.5% (w/v) SDS-polyacrylamide gels (SDS-PAGE) [86] and transferred to nitrocellulose membranes as described [87]. Antibodies raised against FNR (1∶3000; a kind gift from G. Unden, Mainz, Germany), PflB (1∶3000), Hyd-2 (1∶20,000; a kind gift of F. Sargent, Dundee, Scotland), HycG (1∶3000; [88]), NAR (1∶3000) and FDH-N (1∶3000) were used. Secondary antibody conjugated to horseradish peroxidase was obtained from Bio-Rad. Visualisation was done by the enhanced chemiluminescent reaction (Stratagene). Detection of hydrogenase enzyme activity after non-denaturing PAGE (native-PAGE) was performed as described [27]. Gels for hydrogenase activity-staining were loaded with 25 µg protein per lane. In-gel activity of FDH-O was determined using 50 mM sodium formate and 978 mM nitroblue tetrazolium (NBT) as described [59].

### Bioinformatic analysis of BL21(DE3) and MG1655 genome

The global alignment of the analysed gene products against all translated potential open reading frames (ORF) in BL21(DE3) was based on an *ad hoc* implementation of the Needleman-Wunsch algorithm. The scoring function used in this implementation was chosen in such a way that it resulted in a similarity score being equal to the length of the protein in MG1655, if the translated potential ORF in BL21(DE3) matched the protein exactly. This indicated the existence of the respective gene product in BL21(DE3). If for a given protein in MG1655 no exact match was detected in any translated potential ORF in BL21(DE3), the maximal similarity score out of all calculated global alignments was chosen.

## Supporting Information

Figure S1 **Partial complementation of nickel transport- and hydrogenase maturation-defective mutants.** Shown is an activity-stained gel after non-denaturing PAGE analysis of extracts derived from the indicated strains, which were grown anaerobically as described in the methods section of the main text. The locations of Hyd-1 and Hyd-2 are indicated as is a hydrogen-independent activity band (*) frequently observed under these growth conditions. D, original mutant without addition; Ni, growth in the presence of 0.5 mM NiCl_2_; growth of the mutant after transformation with a plasmid carrying the gene that is deleted from the chromosome in the respective mutant (See Table 3 of main text). Strains JW3441 (*nikA*), JW2444 (*nikD*), JW3445 (*nikE*), JW2961 (*hybD*) and JW5493 (*hybF*) were described in [35].
(TIF)

Table S1 **Amino acid exchanges in BL21(DE3) gene products compared to MG1655 with a function in hydrogen metabolism.** * see [92]
(DOCX)

Table S2 **Phenotypic analysis of hydrogen metabolism in different of BL21(DE3) derivatives.** ^1^ Cells were grown in TGYEP pH 6.5. Values in parenthesis were obtained after growth of cells in the presence of 1 mM sodium molybdate. ^2^ The mean and standard deviation of three independent experiments are shown. ^3^ Gas production was measured qualitatively with inverted Durham tubes.
(DOCX)
