# Supplementary figures and images for: Metabolic Deficiences Revealed in the Biotechnologically Important Model Bacterium Escherichia coli BL21(DE3)
Source: PLoS One. 2011 Aug 3;6(8):e22830. doi: 10.1371/journal.pone.0022830 (PMC3149613; doi:10.1371/journal.pone.0022830)

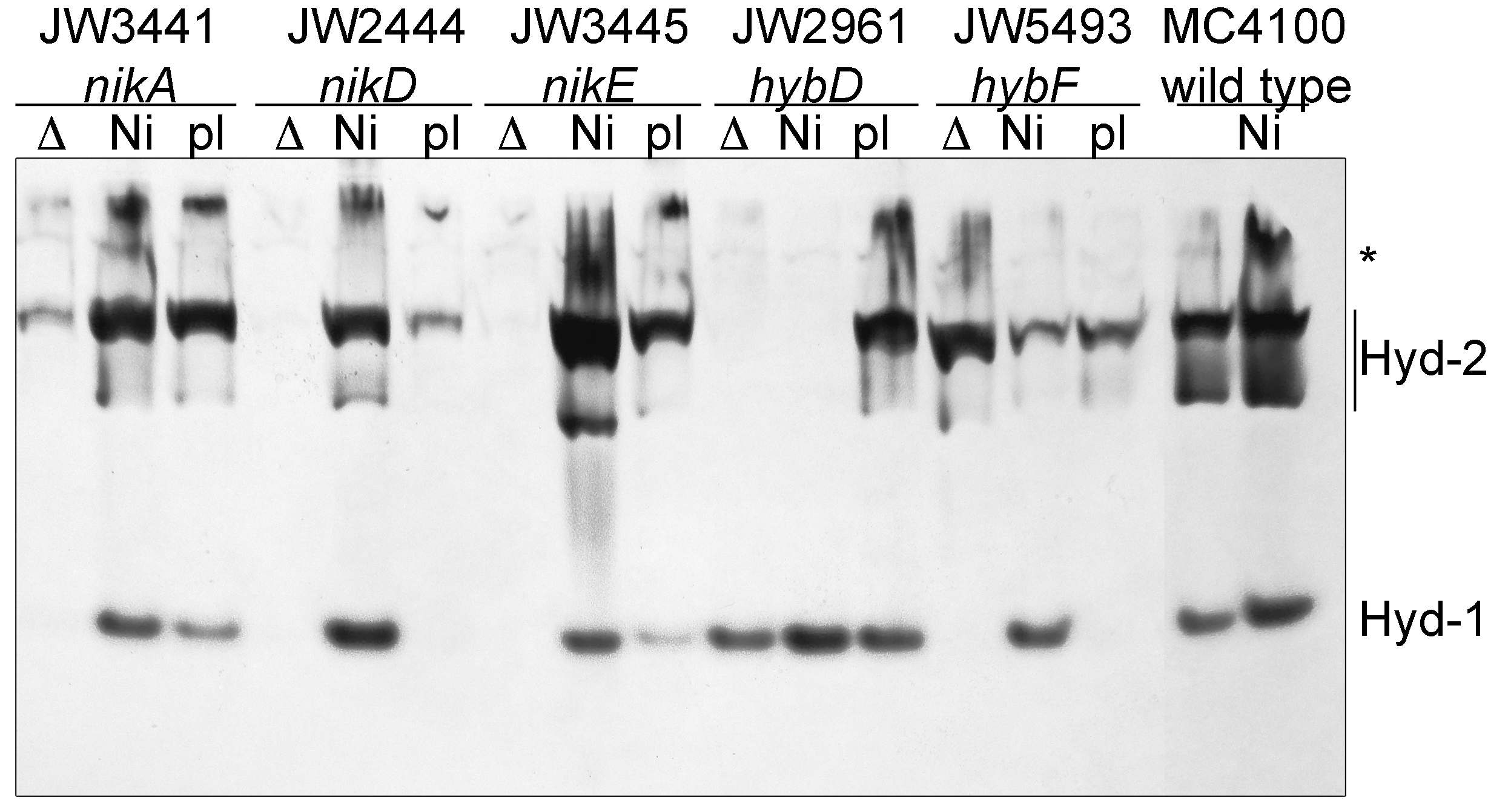

Supplement: Figure S1 — Partial complementation of nickel transport- and hydrogenase maturation-defective mutants. Shown is an activity-stained gel after non-denaturing PAGE analysis of extracts derived from the indicated strains, which were grown anaerobically as described in the methods section of the main text. The locations of Hyd-1 and Hyd-2 are indicated as is a hydrogen-independent activity band (*) frequently observed under these growth conditions. D, original mutant without addition; Ni, growth in the presence of 0.5 mM NiCl2; growth of the mutant after transformation with a plasmid carrying the gene that is deleted from the chromosome in the respective mutant (See Table 3 of main text). Strains JW3441 (nikA), JW2444 (nikD), JW3445 (nikE), JW2961 (hybD) and JW5493 (hybF) were described in [35]. (TIF) [file pone.0022830.s001.tif]
