# Supplementary material for: Metabolic Deficiences Revealed in the Biotechnologically Important Model Bacterium Escherichia coli BL21(DE3)
Source: PLoS One. 2011 Aug 3;6(8):e22830. doi: 10.1371/journal.pone.0022830 (PMC3149613; doi:10.1371/journal.pone.0022830)
Supplement: Table S1 — Amino acid exchanges in BL21(DE3) gene products compared to MG1655 with a function in hydrogen metabolism. * see [92] (DOCX) [file pone.0022830.s002.docx]

Table S1: Amino acid exchanges in BL21(DE3) gene products compared to MG1655 with a function in hydrogen metabolism.

| Protein | Amino acid exchanges |
| --- | --- |
| CarB (carbamoyl phosphate synthetase) | D434E/D487E |
| FdhH (*fdhF* – formate dehydrogenase H) | P551L |
| FNR (transcriptional dual regulator) | Q141Stop |
| HybD (maturation peptidase for Hyd 2) | A70T |
| HybF (maturation of Hyd 1 and 2) | L91I |
| HycA (regulator of the transcriptional regulator FhlA) | T132I |
| HycB (Fe-S subunit of the FHL complex) | A138T |
| HycC (membrane subunit of the FHL complex) | V280M |
| HycD (membrane subunit of the FHL complex) | I46M/I47F |
| HycF (Fe-S subunit of the FHL complex) | H74R |
| HycG (small subunit of Hyd-3) | L175Q |
| HypF (Hyd maturation protein) | R51L/Y62H/K214N/D258E/S565P |
| NikA (periplasmic binding protein of the nickel ABC transporter) | E191A/S330R |
| NikD (ATP-binding component of the nickel ABC transporter) | D216Q |
| NikE (ATP-binding component of the nickel ABC transporter) | I6V/S7C/N19S/A65S/E238D/T265S |
| SelB (Elongation factor for selenocysteine insertion) | M154I/N232H/A316V/F414S |
| SelD (Selenophosphate synthase) | E197D |
| AckA, AdhE, ArcA, CarA, ErpA, FhlA, HyaA-F, HybA-E, HybG, HybO, HycE, HycH, HycI, HypA-E, HydN, IhfA, IhfB, IscA, IscR, NarL, NikB, NikC, NikR, PflAB, PTA, SelA, *selC* (tRNA^Sec^), FdoGHI | None |
| Proteins of hydrogenase 4 operon, usually not synthesized under the conditions tested* | FocB T75A; HyfA D121N/A126V/P127L/T128P; HyfB P66T/I398L/N508D/A512V/Q528R/G571S/A616V; HyfC frameshift at position 289 within gene; HyfD signal peptide altered; HyfE none; HyfF none; HyfG none; HyfH A45-/C46-/G73R/P126Q/I130V/A134T/I167L/L174P; HyfJ A137T; HyfI R24Q/V69I; HyfR S114P/V177A/C221N/ E225D |
| Molybdopterin cofactor biosynthesis | MoaA R61S/S111N  MoaB R22C  MoaC none  MoaD M35L  MoaE N48K/A102E  MobA V1M  MobB none  MogA E150A  MoeA G107V/M120T/A128V/E394D  MoeB I36V/T95A/M211L |
| ModE; ModF; ModA, ModB, ModC (Molybdate transport) | Corresponding genes absent from the genome of BL21(DE3) |
| FdhD (Formate-dehydrogenase N accessory protein) | D152V |
| FdhE (Formate dehydrogenase accessory protein) | D259G |
| Formate dehydrogenase N | FdnG L342F/ S666A  FdnH S249A  FdnI none |
| Nitrate reductase | NarG Q1083P  NarH D298E  NarI P36S/ T52A/ A122S/ L157M |
| RpoN (sigma 54 factor, subunit of RNA polymerase) | E150D/I165M |
