# Supplementary material for: Metabolic Deficiences Revealed in the Biotechnologically Important Model Bacterium Escherichia coli BL21(DE3)
Source: PLoS One. 2011 Aug 3;6(8):e22830. doi: 10.1371/journal.pone.0022830 (PMC3149613; doi:10.1371/journal.pone.0022830)
Supplement: Table S2 — Phenotypic analysis of hydrogen metabolism in different of BL21(DE3) derivatives. 1 Cells were grown in TGYEP pH 6.5. Values in parenthesis were obtained after growth of cells in the presence of 1 mM sodium molybdate. 2 The mean and standard deviation of three independent experiments are shown. 3 Gas production was measured qualitatively with inverted Durham tubes. (DOCX) [file pone.0022830.s003.docx]

**Table S2: Phenotypic analysis of hydrogen metabolism in different of BL21(DE3) derivatives.**

| Strain/Growth condition^1^ | Specific hydrogenase activity in U mg protein^-1^ ± SD | Qualitative H_2_ production^3^ | Hyd-1 and Hyd-2 activity after activity staining of native PAGE |
| --- | --- | --- | --- |
| Rosetta(DE3) pLysS | < 0.01 (<0.01) | - (-) | - (-) |
| Rosetta(DE3) pLysS/pCH21 | 0.07 ± 0.02 (2.51 ± 1.17) | - (+) | + (+) |
| C41(DE3) | < 0.01 (< 0.01) | - (-) | - (-) |
| C41(DE3)/pCH21 | 0.08 ± 0.03 (3.37 ± 0.44) | - (+) | + (+) |
